# Chorion Alterations in Eyed-Stage Salmonid Eggs Farmed in La Araucanía, Chile: A Retrospective Study

**DOI:** 10.3390/ani11082427

**Published:** 2021-08-18

**Authors:** Iván Valdebenito, Elías Figueroa, Matías Valdebenito, Luis Paiva

**Affiliations:** 1 Departamento de Cs. Agropecuarias y Acuícolas, Facultad de Recursos Naturales, Universidad Católica de Temuco, Temuco 4781312, Chile; ivisler@uct.cl (I.V.); efigueroa@uct.cl (E.F.); matias.valdebenito.aguila@gmail.com (M.V.); 2 Núcleo de Investigación en Producción Alimentaria (NIPA), Universidad Católica de Temuco, Temuco 4781312, Chile

**Keywords:** fish embryo, zona pellucida, egg disease, egg quality, broodstock

## Abstract

**Simple Summary:**

The chorion (also called egg envelope) is the primary envelope that protects the fish embryo against drying, mechanical actions, and abrupt changes in the water conditions. Alterations of the chorion during the embryo incubation are not unusual, but these are scarcely reported. Increased occurrence of chorion alterations can lead to decreased reproductive performance and important losses for fish farms. Here, we described several chorion alterations observed in samples of embryonated eggs collected from different salmon and trout farms located in southern Chile over a period of 14 years. We detected four types of chorion alterations and found soft chorion as the most prevalent alteration in the years analyzed, affecting mainly Atlantic and Coho Salmon. Eggs of Rainbow Trout displayed fewer chorion alterations among the three species analyzed. As the eggs analyzed here were produced under standard industrial conditions, we conclude that these alterations are possibly linked to changes in water conditions, which need to be further investigated.

**Abstract:**

The chorion is the primary envelop that protects the fish embryo against mechanical actions, pathogens, and abrupt changes in physical and chemicals conditions of the incubation medium. During embryo development, chorion alterations are not rare, but the occurrence of these is scarcely reported. Increased frequency of chorion alterations can result in increased embryo mortality and thus decreased reproductive performance and losses for fish farms. In this study, we characterize different chorion alterations observed in samples collected over 14 years from 12 salmon and trout farms located in the region of La Araucanía in southern Chile, which sent live eyed-stage embryos (‘eyed-eggs’) for quality analysis to our laboratory. We found soft chorion as the most common alteration observed, being present in the whole 14-year series analyzed in Atlantic Salmon (*Salmo salar*) and affecting up to 35.0% of the samples examined in a year. This alteration also affected up to 20.0 and 5.7% of Coho Salmon (*Oncorhynchus kisutch*) and Rainbow Trout (*Oncorhynchus mykiss*) samples analyzed in a year, respectively. We also found an increase of other chorion alterations, including perforated and white-spotted chorion in Atlantic and Coho Salmon, in the last 8 years. Among the three species, Rainbow Trout exhibited fewer chorion alterations. As the embryonated eggs analyzed here were obtained from broodstocks maintained under standard industrial conditions, these alterations might be linked to changes in environmental conditions affecting the incubation water that need to be further investigated.

## 1. Introduction

The chorion (also called egg envelope) is the primary envelope of the embryo, which is formed by proteins and glycoproteins synthesized in the fish liver, but also by the oocyte itself in some fish species [1,2]. Four distinct glycoproteins are reported to constitute the fish chorion [3], which in Atlantic Salmon (*Salmo salar*) and Rainbow Trout (*Oncorhynchus mykiss*) exhibit molecular weights ranging from 31 to 129 KDa [4,5]. The chorion is organized in three laminae: the outer laminae are homogenous, while the inner lamina has alternating light and dark bands with a soft appearance [6]. The thickness of the chorion varies in different salmonid species ranging from 30 to 60 μm [7,8]; the outermost lamina (externus) exhibits a thickness of 0.3 μm, the lamina (internus) beneath the outer ranges between 25 to 50 μm, and the inner lamina (subinternus) exhibits a thickness ranging between 3 to 8 μm [7].

Once a (single) sperm activates the egg of salmonids, the egg absorbs water, the perivitelline space is formed, and the zona pellucida is modified to form the chorion that hardens. In Atlantic Salmon, the egg permeability is maximal during the hardening process, but then permeability decreases until the eyed-stage embryo [3]. The main function of the chorion is the protection of the embryo against mechanical actions, drying, and abrupt changes in the physicochemical conditions of the milieu, but it is also involved in nutrients uptake, fertilization, and control of pathogen agents [2,9].

Although different parameters have been associated with egg quality [10], several authors [11,12] consider chorion characteristics (e.g., chorion hardness) as one of the important determinants related to egg quality. During embryo development, chorion alterations are not rare, but these alterations are scarcely reported in the literature. Chorion alteration is linked to decreased embryo survival rates [13], and so can result in decreased reproductive performance and important losses for fish farms, for instance, during egg sorting. Thus, it is important to characterize and report the occurrence of these alterations that may be the consequence of variations of intrinsic (e.g., genetic and physiological) and extrinsic (e.g., environmental) factors.

In this study, we characterize chorion alterations observed in eyed-stage embryos (hereafter called ‘eyed-eggs’) of three salmonid fish species intensively farmed in southern Chile that were submitted for egg quality analysis over a 14-year period.

## 2. Materials and Methods

Samples from Atlantic Salmon (*n* = 124), Coho Salmon (*Oncorhynchus kisutch*; *n* = 117), and Rainbow Trout (*n* = 86) females were used in this study. Twenty (20) eyed-eggs per female were sampled from randomly chosen animals and transported alive at 4 °C in the absence of light to the Fish Reproduction Laboratory of the Universidad Católica de Temuco, Temuco, Chile for egg quality analysis over a period of 14 years (2006–2019). These eyed-eggs were incubated and sent from 12 different fish farms located in the mountain range of La Araucanía (between 38°40′ S and 39°40′ S latitude; ~300–650 m above sea level) in southern Chile, which were supplied with waters from the basins of the Allipén, Cautín, Curaco, Trancura, and Toltén rivers. 

The broodstocks were maintained under standard industrial conditions, i.e., kept in gray fiberglass tanks with open flow (water exchange rate of 2 times/h), natural photoperiod and thermoperiod (16 h light/18 °C and 10 h light/8 °C in summer and winter, respectively), and fed with commercial diets given in a range from 0.5 to 1.0% of body weight/day. Animal care and handling were carried out in accordance with the Chilean Animal Protection Act (2009) and regulations governing aquaculture and animal welfare; this research only involved the use of embryonated eggs until eyed-stage of development, which are not considered protected animals (until they reach feeding larval forms) and so no ethical approval was required. 

After spawning, the fertilized eggs of each female were incubated separately in both vertical flow (hatching jar) and horizontal trays incubation systems under standard industrial protocols: 8 to 12 L/min water flow, 5 to 8 °C temperature, absence of light, and without mechanical actions until embryos reach eyed-stage (salmon: 340 accumulated thermal units [ATU]; trout: 250 ATU). Then, eggs were given a mechanical shock (‘shocking’) to separate dead from live eggs, and from the latter, samples were taken and submitted for analysis as indicated above. 

Images were taken using a stereomicroscope (Olympus SMZ-2T; Olympus Co., Tokyo, Japan) attached with an upright digital camera (MicroPublisher 3.3 RTV; Q Imaging, Surrey, BC, Canada) controlled by a PC running the software Q-Capture Pro 5.1 (Q Imaging, Surrey, BC, Canada), which was also used to measure the diameter of eyed-eggs and morphometric parameters for the characterization of chorion alterations. For the determination of the chorion strength, we used a modified push–pull force gauge as described by Kashiwagi et al. [14]; a fixed value of 100 g was applied and flattened or invaginated eyed-eggs after the pressure application were categorized as soft chorion.

We also determined the occurrence of chorion alterations observed from the samples submitted for quality analysis between the years 2006 to 2019. These were calculated as the percentage of eyed-eggs exhibiting a particular chorion alteration from each female analyzed, then these values were averaged in each year for each species. The values are expressed as mean ± SEM.; as this report used (non-experimental) samples from fish farms with different genetic backgrounds and water supplies, no comparisons were made and so only descriptive statistics were used in this study.

## 3. Results

**Figure 1:**
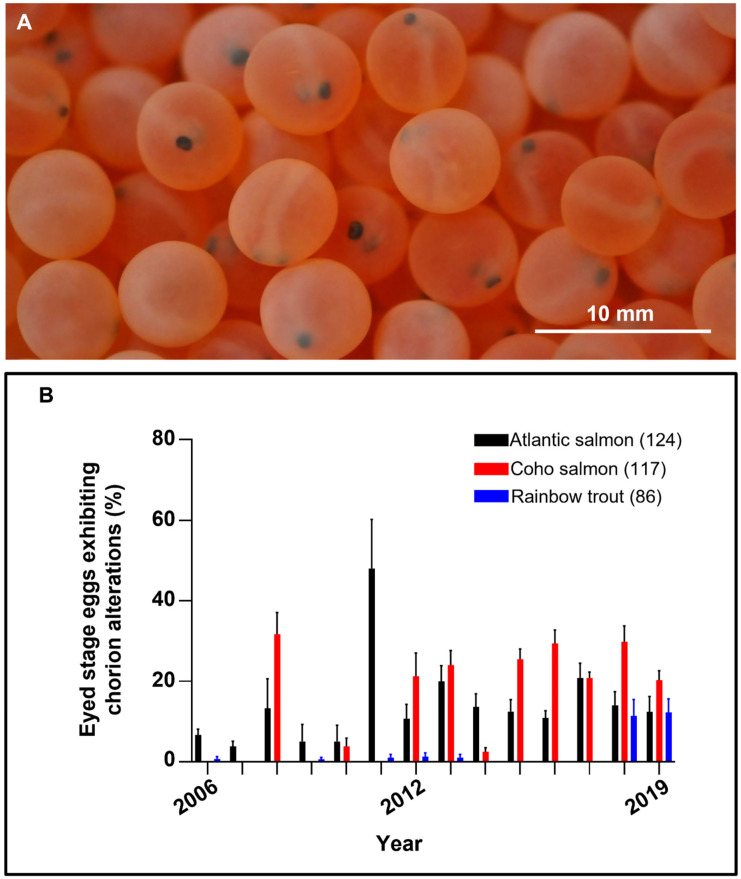
Occurrence of altered chorion in eyed-eggs. (**A**) Representative example of live (Coho Salmon) eyed-stage embryos after mechanical shocking and removal of dead and infertile eggs in which the (**B**) occurrence of chorion alterations were analyzed. Data are means + SEM. Total number of females analyzed in the year series in brackets; twenty eggs per female were analyzed.

In the three fish species analyzed, embryonated eggs exhibiting both normal and altered chorion were in a similar developmental (eyed) stage (Figure 1A). In these eyed-eggs, the mean ± SEM diameter measured in Atlantic Salmon, Coho Salmon, and Rainbow Trout was 5.9 ± 0.05 mm, 6.4 ± 0.04 mm, and 5.1 ± 0.03 mm, respectively. Among the samples examined, chorion alterations arose in variable percentages on the different species in the years analyzed (Figure 1B).

### 3.1. Chorion Alterations

**Figure 2:**
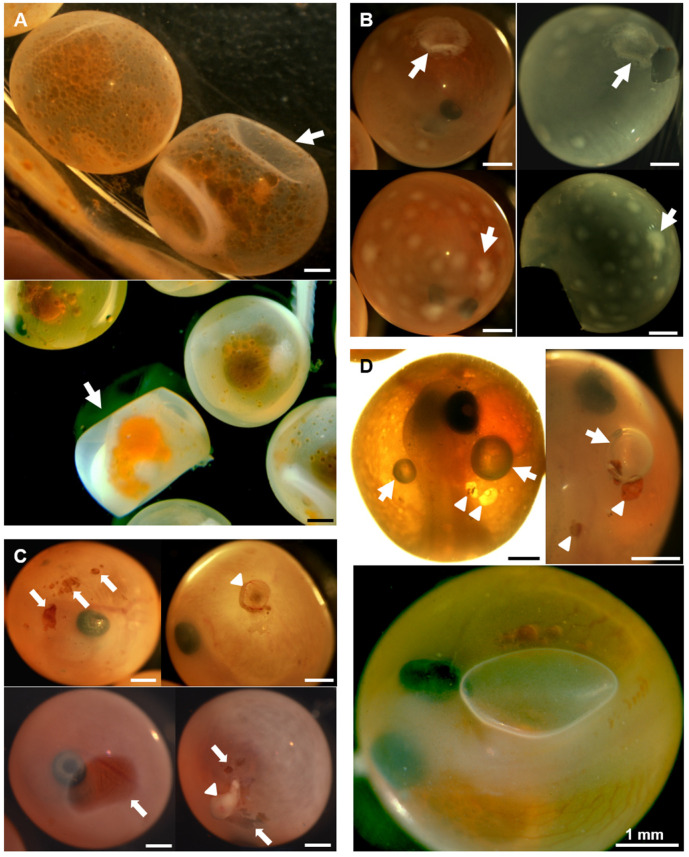
Chorion alterations: (**A**) Soft chorion. Representative examples of normal and soft chorion (arrows) in Atlantic Salmon (upper panel), and Rainbow Trout (lower panel) eyed-eggs. (**B**) White-spotted chorion. White spots (arrows) on chorion before (left panel) and after (right panel) chorion removal in Atlantic Salmon eyed-eggs. (**C**) Perforated chorion. Representative examples of eyed-eggs displaying variable size holes (arrows) and extrusion of the yolk sac (arrowhead; right panel) in Atlantic (upper panel) and Coho (lower panel) Salmon. (**D**) Bubbles in eyed-eggs. Example of Atlantic Salmon (upper panel) eyed-eggs displaying holes (arrowheads) and unidentified gas bubbles (arrows) in the perivitelline space. Rainbow Trout (lower panel) eyed-egg exhibiting a large gas bubble.

The different chorion alterations found exhibited similar characteristic in the three species and based on the appearance of the eyed-eggs can be described as follows: (i) soft chorion (also known as soft egg or soft shell disease), which exhibited a soft texture and low resistance to the pressure that resulted in chorion invagination (Figure 2A); (ii) white-spotted chorion exhibited white spots on the surface of different color intensity and size (ranging from 10 to 1500 µm), these spots remained on the chorion after removal without affecting the embryo itself (Figure 2B); (iii) dark chorion displayed an opaque appearance and embryos could not be visualized, but following chorion removal, the embryos looked normal in appearance. This alteration was exclusively found in Atlantic Salmon in 2006; (iv) perforated chorion showed regular or irregular holes, from which embryos were clearly seen (Figure 2C). These holes ranged between 5 to 2000 µm, and after a soft pressure using forceps, the yolk sac protruded (Figure 2C) resulting in the death of the embryo. We also observed gas bubbles located in the perivitelline space of some eyed-eggs that ranged in size from 50 to 1000 µm, these bubbles appeared mostly in (approximately 1.5% of the) perforated Atlantic Salmon eyed-eggs and some trout eyed-eggs (Figure 2D) that, in some cases, floated.

### 3.2. Occurrence of Chorion Alterations

#### 3.2.1. Atlantic Salmon

In this species, four chorion alterations were identified and quantified whose detection varied among the years; Table 1 shows the detailed percentage of occurrence of these alterations (summarized in Figure 1B). The most common alteration observed in this species was soft chorion, being detected in the whole year series analyzed. This alteration peaked in 2011 with 35.0 ± 11.0% of the samples displaying this disease. White-spotted chorion was detected in 6 years over the last 9 years, the highest occurrence rate (13.0 ± 4.9%) was also observed in 2011. Perforated chorion was observed in 5 years over the last 7 years, peaking in 2017 with 10.0 ± 3.2% of the samples exhibiting it. Dark chorion was observed once in the year series analyzed.

#### 3.2.2. Coho Salmon

Three chorion alterations were identified in this species, whose occurrence was prominent since 2012 (Table 1). White-spotted chorion was the alteration exhibiting the highest frequency of occurrence (8 out of 14 years) over the years analyzed and peaked in 2008 with 31.7 ± 6.7% of the samples displaying it. Soft chorion was detected in 7 years over the last 8 years; the highest rate (20.0 ± 3.5%) was observed in 2013. Perforated chorion was observed in 6 years over the last 8 years, peaking in 2012 with 11.3 ± 3.1% of the samples exhibiting this alteration. Dark chorion was not observed in this species.

#### 3.2.3. Rainbow Trout

Two chorion alterations were observed in this species (Table 1). Soft chorion occurred intermittently over the year series analyzed, arising at a low rate which peaked in 2018 with 5.7 ± 2.5% of the eggs exhibiting it. Perforated chorion was observed in 2018 and 2019 exhibiting a frequency of 5.7 ± 2.8% and 7.3 ± 2.1%, respectively.

## 4. Discussion

Recently, attention has been given to the gradual increase of salmon diseases associated with intensive farming in Chile [15], but little or no attention to egg and embryo alterations occurring in salmonids. The capacity to support embryo development until the hatching of fish larvae is closely linked to egg quality in fish [16], and so chorion abnormality can result in increased embryo mortality. In the present study, we characterize different morphological alterations of the chorion and found soft chorion as the most prevalent alteration occurring in farmed salmonid species.

A previous study [17] has suggested that eggs of Coho and Chinook Salmon (*Oncorhynchus tshawytscha*) farmed in British Columbia are more prone to suffer soft egg disease when incubation temperatures increase (which rise the hydrostatic pressure of eggs, opening chorion pores that would allow the entry of pathogens). Increased occurrence of soft egg disease has also been linked to maternal factors, including ‘dietary stress’ (i.e., limited food supply and dietary deficiencies) during vitellogenesis in Chinook Salmon of the Lake Oahe [13]. In the present study, Atlantic, but not Coho, Salmon was predominantly affected by soft egg (chorion) disease in fish farms located in southern Chile; an increase in the occurrence of this alteration was observed in Coho Salmon —and to a lesser extent in Rainbow Trout— in the last 8 years. As the eyed-eggs analyzed in this study were taken from broodstocks fed with balanced commercial diets, increased or fluctuating incubation temperatures seem to be a more obvious factor related to this disease.

Following fertilization, egg hardening is a process that involves calcium, chorion protein modifications, and water uptake [18]. In intensive systems, disinfection treatments are routinely applied after egg hardening (i.e., fertilization) to reduce pathogens, but it is known that some chemical agents used for this purpose may have deleterious effects on chorion in a dose-dependent fashion. Kashiwagi et al. [14] have reported that sodium hypochlorite applied after fertilization at 30 mg/L for 60-min every third day, but not 10 mg/L for 15-min daily, induces egg softening in Rainbow Trout. In Chile, the national regulatory agency (SERNAPESCA) dictates the compulsory use of (available) iodine given at a concentration of, at least, 100 ppm for 10-min, pH 6–8, for the disinfection of farmed egg. This dose has been previously shown to be harmless for the survival of eyed-eggs [19,20].

In this study, other chorion alterations became prominent in the last years; white-spotted chorion was observed in both Coho and Atlantic Salmon, with no cases in Rainbow Trout. Of note is the resistance of Rainbow Trout’s chorion to suffer alterations, a plausible explanation might be related to the chorion thickness which, in the case of Atlantic Salmon, thick chorion (42 µm) has been linked to better protection against Saprolegnia infections than thin chorion (35 µm) [21]; data from other studies indicate that the eggs of Rainbow Trout exhibit the thickest chorion (46.4 µm [22]) when compared to Coho (27.96 µm [7]) and Atlantic (30.5 µm [23]) Salmon. Saprolegniasis is a fungal infection commonly found in fish and eggs of salmonids maintained in freshwater [24], and when it develops it is known for its cotton-like appearance on the surface of eggs, turning these white in appearance [25]. Nevertheless, we did not find compatible structures attaching on the surface of eyed-eggs, indicating that white spot alteration affects the chorion layers themselves, possibly by focal alterations of the chorion ultrastructure as reported in hard eggs [23].

We also found the occurrence of perforated chorion, and this alteration has been observed in both the University laboratory setting and fish farms in situ, and so it was not induced by friction during transport of samples, which involved ~1.5 h transport over mainly asphalted roads. Chorion perforation has been associated with an advance of embryo hatching, which is induced by mechanical and enzymatic effects. In teleost fish, eyed-stage embryos begin to secrete proteases that digest the chorion, commonly called hatching enzymes [26] and, in mature eggs of Atlantic Salmon, premature hatching has been shown to be induced by several experimental conditions including hypoxia, alkaline pH, and neurotransmitter administration [27]. Conversely, Atlantic Salmon eyed-eggs exposed to water with low pH (>5.5) exhibited delayed hatch [28]. Interestingly, the environmental conditions in which broodstocks are maintained is reported to affect the chorion breaking strength and composition, and consequently, embryo survival [12].

Unfortunately, there are limited reports characterizing the occurrence of different egg diseases in salmonids, perhaps because data records are confidential at salmon farming companies, making it difficult to compare the observations presented here with data originating from other geographical locations. Similarly, data regarding physicochemical and quality analyses of the water sources used to hatch the embryonated eggs analyzed here remain unknown, possibly for similar reasons. Parameters including oxygen [27] and temperature [17] have been shown to affect the attributes of fertilized eggs during the hatch and are also likely to affect pathogens load in the incubation milieu, which is thought to play a role in eggs diseases. Furthermore, in recent years, environmental issues have been suggested to affect salmon farming in Chile [29]; in particular, agricultural and aquaculture activities upstream have resulted in an increase of dissolved organic matter that leads to a rise in bacterial load [30] and also increased content of nitrogen and phosphorus [31] in rivers of southern Chile. Interestingly, an increased presence of ammonium in water [32], and potassium and phosphorus in chorion [12] are linked to chorion alterations. Therefore, environmental changes that affect water quality seems to play a major role by affecting the incubation conditions in which fertilized eggs are maintained and so the occurrence of diseases and alterations in these.

A limitation of this study is the relatively small sample size from which observations were made, and so quantitative data must be interpreted carefully for comparative conclusions. Determining whether water parameters have changed over the years reported here and its potential relationship with the occurrence of chorion alterations is key to take actions to preserve or improve the reproductive performance of salmon farms.

## 5. Conclusions

Soft chorion is the most common and relevant alteration affecting eyed-eggs of salmonid species hatched in La Araucania, Chile; but there is an increase in the occurrence of other chorion alterations affecting salmon and, to a lesser extent, trout eyed-eggs in the last years. As the embryonated eggs analyzed here were obtained from broodstocks maintained under standard industrial conditions, these alterations are possibly linked to changes in environmental conditions affecting the incubation water.

## Figures and Tables

**Table 1:** Occurrence rate (%) of characterized chorion alterations.

Species	Chorion Alteration	Year													
		2006	2007	2008	2009	2010	2011	2012	2013	2014	2015	2016	2017	2018	2019
*S. salar*	Soft	3.3 ± 1.7	3.8 ± 1.3	13.3 ± 7.3	5.0 ± 5.0	5.0 ± 5.0	35.0 ± 11.0	10.7 ± 3.8	15.0 ± 3.1	10.0 ± 3.6	5.0 ± 1.7	0.5 ± 0.5	10.0 ± 1.7	6.0 ± 2.1	4.3 ± 1.7
	Perforated								5.0 ± 1.9		7.5 ± 3.0		10.0 ± 3.2	2.0 ± 1.0	4.8 ± 2.6
	White-Spotted						13.0 ± 4.9			3.6 ± 2.8		10.5 ± 1.7	0.8 ± 0.8	6.0 ± 2.8	3.3 ± 2.0
	Dark	3.3 ± 1.7													
	*n*	7	5	5	5	6	5	7	7	7	10	11	13	15	21
*O. kisutch*	Soft							10.0 ± 3.5	20.0 ± 3.5		11.7 ± 1.7	10.6 ± 0.6	13.1 ± 0.9	10.0 ± 1.8	7.4 ± 1.2
	Perforated							11.3 ± 3.1	4.0 ± 1.0			7.5 ± 1.6	6.2 ± 1.2	10.7 ± 2.3	5.2 ± 1.4
	White-Spotted			31.7 ± 6.7		3.8 ± 2.4				2.5 ± 1.1	15.0 ± 2.2	11.3 ± 2.1	1.5 ± 0.9	9.1 ± 1.8	7.6 ± 1.3
	Dark														
	*n*	5	5	6	5	5	7	5	5	6	10	8	13	16	21
*O. mykiss*	Soft	0.7 ± 0.7			0.6 ± 0.6		1.0 ± 1.0	1.3 ± 1.3	1.0 ± 1.0					5.7 ± 2.5	5.0 ± 2.2
	Perforated													5.7 ± 2.8	7.3 ± 2.1
	White-Spotted														
	Dark														
	*n*	7	5	5	8	5	5	5	5	5	6	5	5	7	13

Data means ± S.E.M. *n* = number of females submitted for analysis; from each female twenty eggs were analyzed.

## Data Availability

Data are contained within the article.
